# Serum predictors of native liver survival post‐Kasai: Systematic review and meta‐analysis

**DOI:** 10.1002/jpn3.70355

**Published:** 2026-01-29

**Authors:** Ahmad Anouti, Hamza Dahshi, Thomas G. Cotter, Tebyan A. Rabbani, Natasha Corbitt, Sara Hassan

**Affiliations:** ^1^ Department of Pediatrics University of Texas Southwestern Medical Center Dallas Texas USA; ^2^ School of Medicine Case Western Reserve University Cleveland Ohio USA; ^3^ Division of Digestive and Liver Diseases University of Texas Southwestern Medical, Center Dallas Texas USA; ^4^ Division of Gastroenterology, Hepatology, Nutrition Stanford University Stanford California USA; ^5^ Department of Surgery University of Texas Southwestern Medical Center Dallas Texas USA; ^6^ Department of Pediatrics, Division of Gastroenterology, Hepatology and Nutrition University of Texas Southwestern, Children's Health Dallas Texas USA

**Keywords:** biliary atresia (BA), direct bilirubin (DB), hepatoportoenterostomy (HPE), total bilirubin (TB)

## Abstract

**Objectives:**

After hepatoportoenterostomy (HPE), a minority of biliary atresia (BA) patients reach adolescence without liver transplantation. Several serum markers have been suggested to better predict post‐HPE outcomes in BA patients. We aimed to identify serum predictors of native liver survival (NLS) in post‐HPE BA patients.

**Methods:**

We searched PubMed, MEDLINE, SCOPUS, and EMBASE databases to identify publications from 1946 through December 2023. Studies included reported serum values as prognostic factors for BA after HPE, specifically total bilirubin (TB), alanine transaminase (ALT), gamma‐glutamyl transferase (GGT), matrix metalloproteinase 7 (MMP‐7), and total bile acids (TBA). We defined nonfunctioning HPE as persistent jaundice, cirrhosis, LT, or death. We calculated pooled serum variables, including hazard and odds ratios, for NLS using inverse variance weighting.

**Results:**

Thirty studies were included in the meta‐analysis including a total of 4399 BA patients, 2073 and 1793 had successful versus nonfunctioning HPE, respectively. The mean HPE age for the successful group was significantly less (63.3 vs. 69.5 days, *p* < 0.001). TB was significantly elevated in the nonfunctioning group (*p* < 0.001). Pooled ALT and GGT were significantly lower in the successful group 1–3 months and >5 years after HPE (*p* < 0.001). The successful group had significantly lower serum TBA (27.55 vs. 69 μmol/L, *p* < 0.001) > 5 years after HPE.

**Conclusion:**

Established serum values ALT, GGT, and TB are useful for prognostic assessment post‐HPE. MMP‐7 and TBA require additional evaluation to determine their prognostic relevance for NLS in BA.

## Abstract (highlights)

**What is Known:**

- Total bilirubin (TB), alanine transaminase (ALT), and gamma‐glutamyl transferase (GGT) are recognized as prognostic indicators for assessing native liver survival (NLS) in biliary atresia (BA) patients.
- HPE performed at an earlier age, particularly before 60 days of life, significantly improves long‐term NLS outcomes.
- TB levels below 2 mg/dL at 3 months post‐HPE have been consistently identified as a predictor of better prognosis.

**What is New:**

- The study introduces new TB cutoff values, such as <1 mg/dL at 12 months post‐HPE, as a precise prognostic marker for long‐term NLS.
- Matrix metalloproteinase 7 (MMP‐7) and total bile acids (TBA) have emerged as promising, though not yet fully validated, serum markers for predicting NLS.
- A timeline for monitoring serum markers (e.g., TB, ALT, GGT) at 3 months, 6 months, 1 year, and annually thereafter, with tailored cutoff values to improve surveillance and management post‐HPE.

## 1 INTRODUCTION

Biliary atresia (BA), the most common etiology for neonatal cholestasis, is a hepatobiliary disorder characterized by progressive obliteration of bile ducts leading to cirrhosis and hepatic decompensation. Early diagnosis and hepatoportoenterostomy (HPE) surgery to restore bile flow in infants less than 60–90 days old have variable results.^1^, ^2^, ^3^ 87.8% of BA patients in the United States requiring a liver transplant (LT) by less than 12 years of age.^4^

HPE performed in BA infants <30 days old results in improved native liver survival (NLS) at 5, 10, and even 20 years after surgery.^5^ Liver histopathology with severe fibrosis, cholestasis, and lobular inflammation prior to HPE is associated with reduced NLS among BA patients.^6^

Serum bilirubin, alanine transaminase (ALT), and gamma‐glutamyl transferase (GGT) have been used to assess the prognosis of BA patients post‐HPE.^7^ Moreover, additional metrics such as total bile acids (TBA) and the serum marker matrix metalloproteinase 7 (MMP‐7) are emerging as a possible prognostic factor post‐HPE.^8^, ^9^, ^10^

A comprehensive review and meta‐analysis encompassing all studies is warranted to ascertain the relative strength of associations between these prognostic variables and NLS, offering valuable insights with significant clinical implications. Therefore, this meta‐analysis aims to determine the usefulness of serum bilirubin, ALT, GGT, TBA, and MMP‐7 to serve as prognostic tools for NLS in BA patients after HPE.

## 2 METHODS

### 2.1 Ethics statement

Our study conformed to the 1975 Declaration of Helsinki, as revised in 2013 and the 2008 Declaration of Istanbul. This study was reviewed by the institutional IRB and determined to be exempt from the requirement for IRB approval.

### 2.2 Search strategy

We searched Ovid MEDLINE, Ovid MEDLINE In‐Process, Ovid EMBASE, PubMed, and Scopus from 1946 (reflecting the earliest year indexed and searchable in the databases) through December 2023, using search terms described in Supplemental Methods. A manual search of references from relevant articles was also performed to identify publications missed by search terms. A manual search of American College of Gastroenterology, Digestive Diseases Week, American Association for the Study of Liver Diseases (AASLD), European Association for the Study of the Liver, and North American Society for Pediatric Gastroenterology, Hepatology, and Nutrition meeting abstracts was also completed. This study was conducted in accordance with Preferred Reporting Items for Systematic Review and Meta‐analysis (PRISMA) guidelines.^11^

### 2.3 Study selection and inclusion/exclusion criteria

The inclusion criteria were: (1) cohort, cross‐sectional, or case‐control studies reporting original data that characterizes prognostic indicators among BA patients globally, (2) BA patients that have undergone HPE and have either NLS at specific time points (1–3 months, 4–12 months, 1–4 years, and >5 years) or had persistent jaundice defined as TB ≥ 2 mg/dL 3 months post‐HPE, cirrhosis, LT, or death, and (3) reported at least one of the following serum values: total bilirubin (TB), ALT, GGT, MMP‐7, or TBA. We excluded studies that: (1) only reported pre‐HPE serum values, (2) described serum values among the BA population with no comparator or follow‐up, and (3) studies that assessed the use of serum values in detecting post‐HPE complications (e.g. variceal hemorrhage, cholangitis, portal hypertension).

### 2.4 Data extraction and quality assessment

Studies were screened and reviewed in a collaborative, multi‐step process. After removing duplicates, two investigators (A.A. and H.D.) independently reviewed publications identified by the search strategy. Articles were screened based on title and abstract for relevance, followed by full text review to assess for inclusion. Disagreements between authors were resolved by discussion with a third reviewer (S.H.). Using standardized forms, two authors (A.A. and H.D.) independently extracted data including patient demographics (i.e., gender, patient age, and age at HPE), and serum values. Study quality was assessed using a modified checklist based on the NIH Quality Assessment Tool for Observational Cohort and Cross‐sectional Studies, which rates observational studies on a 14‐point scale by two investigators (A.A. and S.H.).^12^

### 2.5 Statistical analysis

For each study, we calculated pooled values and pooled standard errors for prognostic indicators and values of serum TB, ALT, GGT, MMP7, TBA, and age at HPE. When applicable, we stratified study outcomes with comparator groups into “Successful” versus “Non‐functioning” HPE with non‐function indicated by report of persistent jaundice (TB ≥ 2 mg/dL 3 months post‐Kasai), cirrhosis, LT, or death in a BA patient post‐HPE. We further categorized and analyzed the data across four distinct post‐HPE timeframes: 1–3 months, 4–12 months, 1–4 years, and over 5 years NLS. Success and non‐functioning was further stratified by the study time points. Studies with distinct groups of BA patients with successful versus nonfunctioning HPE within these separate timeframes were pooled and analyzed separately. Using the inverse variance weighting method pooled serum values, pooled HR, and pooled OR were calculated. Methods described in Wan et al. were utilized to approximate study estimates and standard errors when a mean and standard error were not available.^13^ When studies reported dichotomized markers (e.g., TB ≥ 2 mg/dL at 3 months), we extracted the reported OR/HR comparing “above” versus “below” the study‐defined cutoff and pooled log‐effects using random‐effects models. For continuous markers, we used study‐reported per‐unit effects; where necessary, we rescaled to common units (TB per 1 mg/dL; GGT per 100 IU/L; ALT per 100 IU/L; TBA per 10 µmol/L) before pooling. For *p*‐values and confidence limits, calculations are based on the standard normal distribution. All statistical tests were two‐tailed. *p* < 0.05 was considered statistically significant. All data analysis was performed using R software (version 4.2.2).

## 3 RESULTS

### 3.1 Literature search

**Figure 1:**
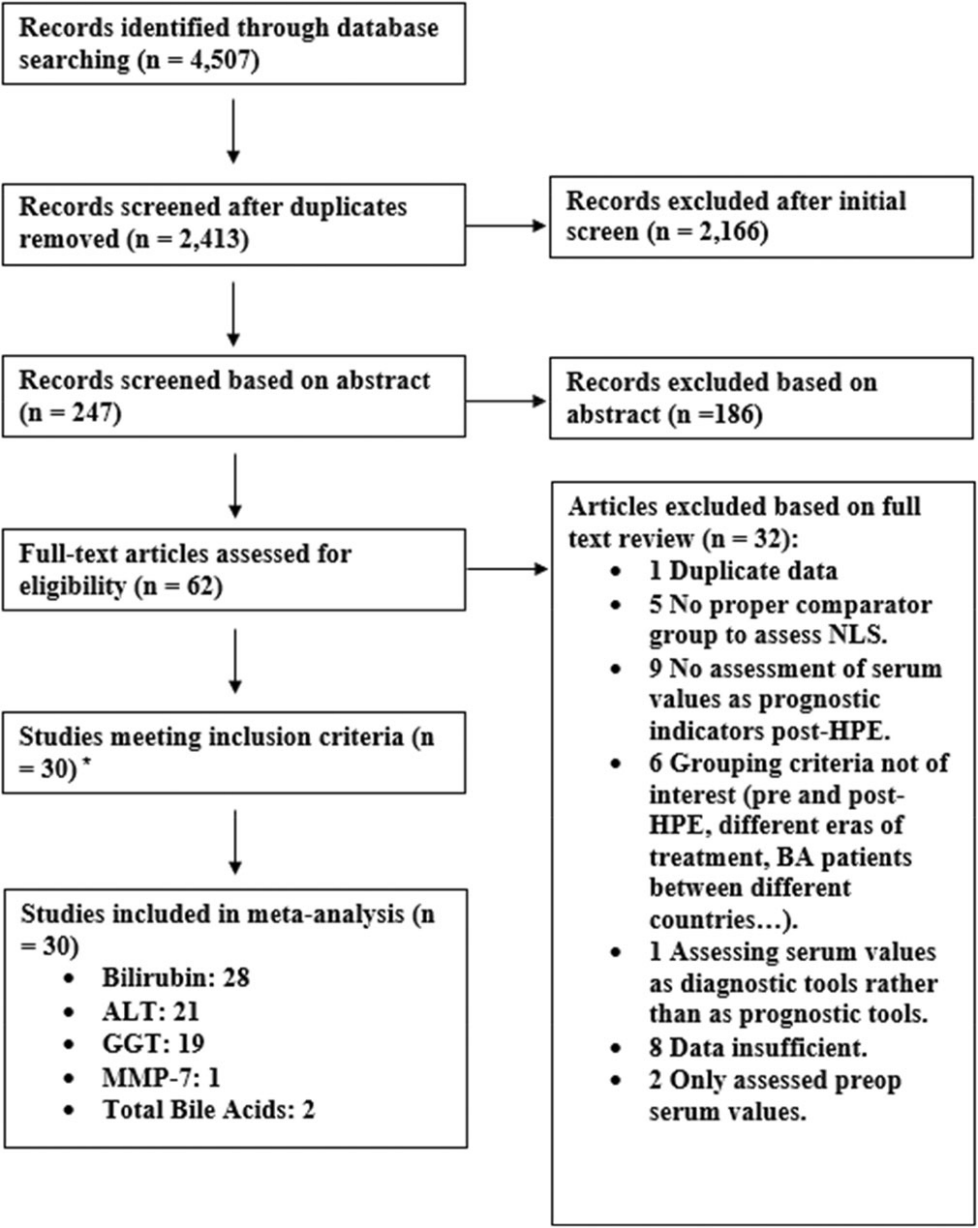
Study flow diagram and search strategy used. Total of papers per serum values > 30 as there is overlap between papers for each value. ALT, alanine transaminase; BA, biliary atresia; GGT, gamma‐glutamyl transferase; HPE, hepatoportoenterostomy; MMP‐7, matrix metalloproteinase 7; NLS, native liver survival.

The search yielded 4507 potentially relevant citations. After removing 2094 duplicate citations, 2413 unique citations remained. Two hundred and forty‐five titles met inclusion criteria. Abstract review of these 245 publications, 63 met inclusion criteria. Of the 63 abstracts, full‐text review resulted in a total of 30 studies meeting inclusion criteria for analysis. Agreement between reviewers for final study inclusion exceeded 95% (Figure 1).

### 3.2 Study characteristics

From 30 studies (Supporting Information S1: Table S1), the total number of BA patients post‐HPE was 4399. A total of 2073 patients were designated to the success group and 1793 to the nonfunctioning group. The mean age of HPE in the success group was 63.3 ± 0.18 days (95% CI: 62.9–63.6) compared to 69.5 ± 0.20 days (95% CI: 69.1–69.9) in the nonfunctioning group (*p* < 0.001). Among 30 studies, 28 reported TB,^14^, ^15^, ^16^, ^17^, ^18^, ^19^, ^20^, ^21^, ^22^, ^23^, ^24^, ^25^, ^26^, ^27^, ^28^, ^29^, ^30^, ^31^, ^32^, ^33^, ^34^, ^35^, ^36^, ^37^, ^38^, ^39^, ^40^, ^41^ 21 reported ALT,^14^, ^16^, ^18^, ^19^, ^20^, ^23^, ^24^, ^25^, ^26^, ^27^, ^28^, ^29^, ^30^, ^31^, ^34^, ^35^, ^36^, ^37^, ^38^, ^41^, ^42^ 19 reported GGT,^14^, ^16^, ^17^, ^19^, ^20^, ^21^, ^22^, ^24^, ^25^, ^26^, ^28^, ^29^, ^30^, ^35^, ^36^, ^38^, ^41^, ^42^, ^43^ 2 reported TBA,^28^, ^30^ and 1 reported MMP‐7^39^ (Table 1 and Supporting Information S1: Table S2).

**Table 1:** Serum values of BA patients included in the overall study.

Serum values	Serum values	1–3 months	4–12 months	1–4 years	>5 years
Total bilirubin (95% CI) mg/dL	TB (Success)	2.36 (2.27–2.45)	0.78 (0.70–0.86)	0.72 (0.67–0.77)	0.25 (0.23–0.27)
	TB (Nonfunction)	6.28 (6.09–6.48)	4.08 (3.81–4.34)	1.52 (1.42–1.62)	2.48 (2.25–2.70)
	*p*‐Value	<0.001	<0.001	<0.001	<0.001
ALT (95% CI) IU/L	ALT (Success)	106.71 (103.66–109.75)	149.89 (139.67–160.11)	116.83 (113.97–119.68)^a^	84.59 (69.87–99.31)
	ALT (Nonfunction)	195.64 (190.27–201.02)	153.43 (150.05–156.82)	121.49 (113.04–129.94)^a^	189.41 (162.32–216.50)
	*p*‐Value	<0.001	0.52	0.31^a^	<0.001
GGT (95% CI) IU/L	GGT (Success)	496.16 (466.50–525.81)	309.94 (277.01–342.87)	338.67 (332.73–344.61)^a^	124.95 (100.53–149.36)
	GGT (Nonfunction)	600.33 (552.88–647.78)	467.99 (459.25–476.75)	363.16 (322.04–404.28)^a^	495.21 (417.52–572.90)
	*p*‐Value	<0.001	<0.001	0.25^a^	<0.001
TBA (µmol/L) (95% CI)	TBA (Success)	n/a	129 (117.10–140.90)	92.47 (75.85–109.095)	27.55 (20.35–34.75)
	TBA (Nonfunction)	n/a	163 (139.44–186.56)	81.75 (71.02–92.48)	69 (56.26–81.74)
	*p*‐Value	n/a	0.01	0.29	<0.001

Abbreviations: Alk Phos, alkaline phosphatase; ALT, alanine transaminase; BA, biliary atresia; CI, confidence interval; GGT, gamma‐glutamyl transferase; TB, total bilirubin; TBA, total bile acid.

^a^ Outlier (Caruso et al.) was excluded.

Further stratification of studies by post‐HPE follow‐up timeframe revealed that 11 studies (*n* = 753) reported outcomes at 1–3 months (326 success, 403 nonfunctioning, 24 unlabeled),^14^, ^17^, ^18^, ^19^, ^20^, ^21^, ^22^, ^23^, ^32^, ^33^, ^34^ 7 studies (*n* = 889) at 4–6 months (485 success, 278 nonfunctioning, 126 unlabeled),^18^, ^22^, ^24^, ^30^, ^31^, ^42^ 16 studies (*n* = 2742) at 1–4 years (1390 success, 1214 nonfunctioning, 138 unlabeled),^15^, ^16^, ^25^, ^27^, ^28^, ^29^, ^30^, ^31^, ^35^, ^36^, ^37^, ^38^, ^39^, ^40^, ^41^, ^42^ and 6 studies (*n* = 778) at >5 years (321 success, 197 nonfunctioning, 260 unlabeled).^18^, ^26^, ^28^, ^30^, ^36^, ^43^ For the 1–3 month group, the pooled mean age at HPE was 68.66 days (95% CI: 67.92–69.39) versus 73.51 days (95% CI: 72.70–74.31) (*p* < 0.01), and for the 1–4 year group, mean HPE age was 61.37 days (95% CI: 60.98–61.77) versus 64.89 days (95% CI: 63.43–66.35) (*p* < 0.01), for the success versus nonfunctioning groups, respectively. For the 4–6 month group, the pooled mean age at HPE was 67.75 days (95% CI: 65.66–69.85) for the success group versus 68.28 days (95% CI: 67.79–68.77) for the nonfunctioning group (*p* = 0.63). As for the >5 year group, the pooled mean age at HPE was 66.28 days (95% CI: 64.31–68.25) for the success group versus 67.21 days (95% CI: 65.22–69.20) for the failure group (*p* = 0.52).

### 3.3 TB

**Figure 2:**
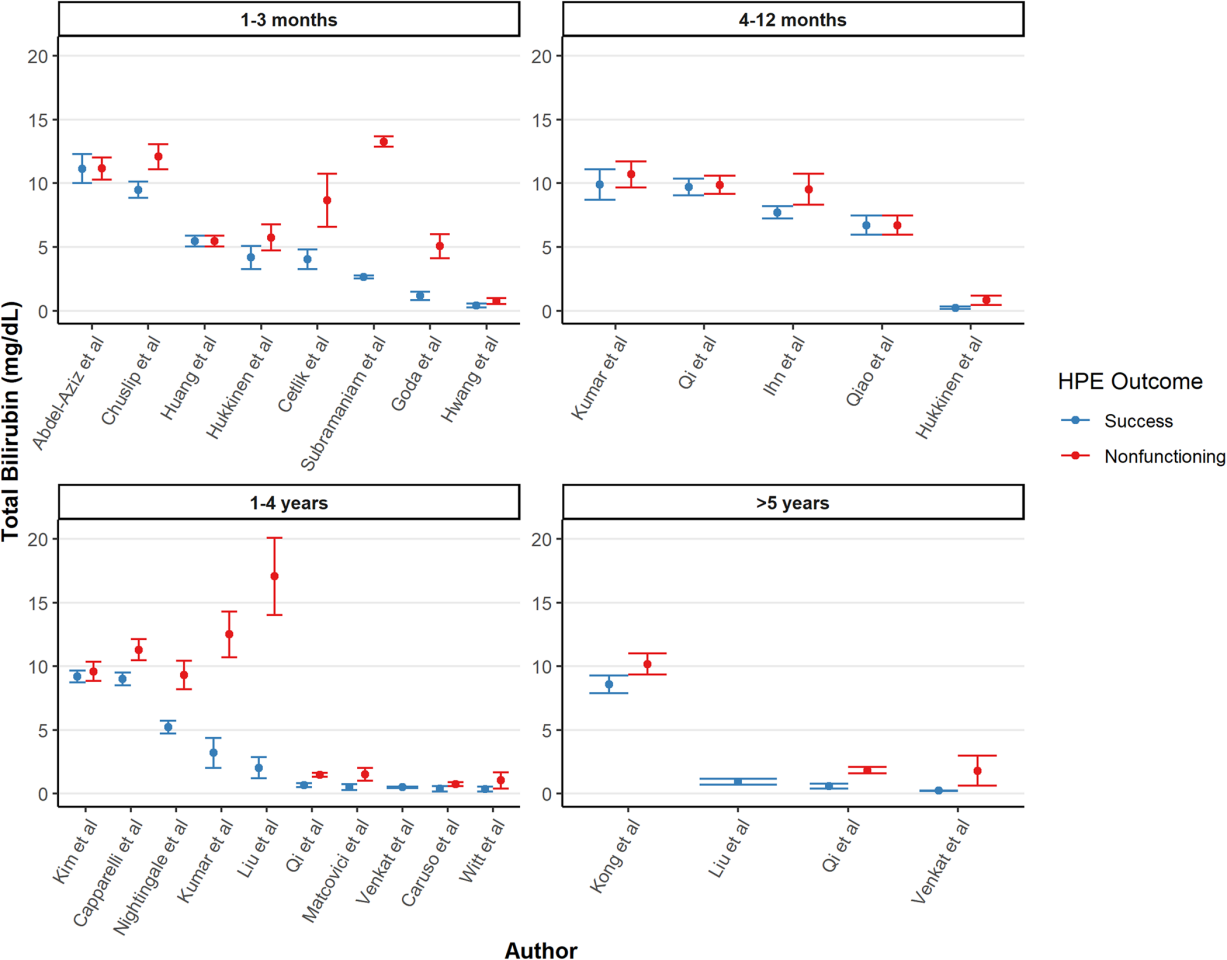
TB (mg/dL) serum values of the studies included stratified by success and non‐function post‐HPE among BA patients. BA, biliary atresia; HPE, hepatoportoenterostomy; TB, total bilirubin.

For the 1–3 month interval after HPE, the pooled serum TB was 2.36 mg/dL (95% CI: 2.27–2.45) for the success group and 6.28 mg/dL (95% CI: 6.09–6.48) for the nonfunctioning group (Figure 2A, *p* < 0.001).^14^, ^15^, ^16^, ^17^, ^18^, ^19^, ^20^, ^21^, ^22^, ^23^, ^24^, ^25^, ^26^, ^27^, ^28^, ^29^, ^30^, ^31^, ^32^, ^33^, ^34^, ^35^, ^36^, ^37^, ^38^, ^39^, ^40^, ^41^ Elevated serum TB at 1–3 months showed an increased pooled odds ratio (OR) of 1.19 (95% CI: 1.09–2.09) for HPE nonfunctioning. For the 4–12 month interval after HPE, the pooled serum TB serum was 0.78 mg/dL (95% CI: 0.70–0.86) for the success group and 4.08 mg/dL (95% CI: 3.81–4.34) for the non‐functioning group (Figure 2B, *p* < 0.001). Elevated serum TB at 4–12 months showed a significantly increased pooled OR of 3.47 (95% CI: 2.15–5.60) for HPE non‐functioning, while the pooled hazards ratio (HR) of 0.94 (95% CI: 0.56–1.57) was insignificant (Supporting Information S1: Figure S1). For the 1‐to‐4‐year interval after HPE, the pooled serum TB was 0.72 mg/dL (95% CI: 0.67–0.77) and 1.52 mg/dL (95% CI: 1.42–1.62) for the successful and nonfunctioning HPE BA populations, respectively (Figure 2C, *p* < 0.001). Elevated serum TB at 1–4 years after HPE showed a significantly increased pooled OR of 1.02 (95% CI: 1.01–1.03) for HPE nonfunctioning, while the pooled HR of 1.00 (95% CI: 0.99–1.01) was insignificant (Supporting Information S1: Figure S1). After more than 5 years' time from HPE, the pooled serum TB was 0.25 mg/dL (95% CI: 0.23–0.27) and 2.48 mg/dL (95% CI: 2.25–2.70) for the successful and nonfunctioning HPE BA populations, respectively (Figure 2D, *p* < 0.001). There was no significant difference in the pooled HR of 0.99 (95% CI: 0.99–1.01) in elevated serum TB more than 5 years after HPE (Supporting Information S1: Figure S1).

### 3.4 ALT

At 1–3 months post‐HPE, the pooled serum ALT was 106.71 IU/L (95% CI: 103.66–109.75) and 195.64 IU/L (95% CI: 190.27–201.02) for the success and nonfunctioning groups, respectively (Supporting Information S1: Figure S2A, *p* < 0.001). At 4–12‐months post‐HPE, pooled serum ALT was 149.89 IU/L (95% CI: 139.67–160.11) and 153.43 IU/L (95% CI: 150.05–156.82) for the success and nonfunctioning groups, respectively (Supporting Information S1: Figure S2B, *p* = 0.52). At 1–4 years post‐HPE, with one outlier by Caruso et al. excluded,^16^ the pooled serum ALT was 116.83 IU/L (95% CI: 113.97–119.68) and 121.49 IU/L (95% CI: 113.04–129.94) for the success and non‐functioning groups, respectively (Supporting Information S1: Figure S2C, *p* = 0.31). Elevated serum ALT at 1–4 years showed a significantly decreased pooled OR: 0.99 (95% CI: 0.98–0.99) for post‐HPE non‐function, while pooled HR: 0.99 (95% CI: 0.99–1.01) was not statistically significant (Supporting Information S1: Figure S3). Greater than 5 years post‐HPE, the pooled serum ALT was 84.59 IU/L (95% CI: 69.87–99.31) and 189.41 IU/L (95% CI: 162.32–216.50) for success and nonfunctioning groups, respectively (Supporting Information S1: Figure S2D, *p* < 0.001).

### 3.5 GGT

At the 1–3 month time point post‐HPE, the pooled serum GGT was 496.16 IU/L (95% CI: 466.50–525.81) and 600.33 IU/L (95% CI: 552.88–647.78) for the success and nonfunctioning groups, respectively (Supporting Information S1: Figure S4A, *p* < 0.001). At the 4–12‐month interval post‐HPE, pooled serum GGT was 309.94 IU/L (95% CI: 277.01–342.87) and 467.99 IU/L (95% CI: 459.25–476.75) for the success and non‐functioning groups, respectively (Supporting Information S1: Figure S4B, *p* < 0.001). Elevated serum GGT at 4–12 months showed an increased pooled OR: 1.74 (95% CI: 1.22–2.48) for HPE nonfunctioning risk (Supporting Information S1: Figure S5). At the 1–4 year interval post‐HPE, with outliers excluded,^16^ the pooled serum GGT was 338.67 IU/L (95% CI: 332.73–344.61) and 363.16 IU/L (95% CI: 322.04–404.28) for the success and non‐functioning groups, respectively (Supporting Information S1: Figure S4C, *p* = 0.25). Elevated serum GGT at 1–4 years post‐HPE showed a significantly increased pooled HR: 1.56 (95% CI: 1.31–1.86) for HPE nonfunctioning. For the >5 years interval post‐HPE, the pooled serum GGT was 124.95 IU/L (95% CI: 100.53–149.36) and 189.41 IU/L (95% CI: 162.32–216.50) for the success and nonfunctioning groups, respectively (Supporting Information S1: Figure S4D, *p* < 0.001).

### 3.6 TBA

At the 1–3 month interval post‐HPE, there were no reported serum TBA values assessed in the literature. At the 4–12 month interval post‐HPE, pooled serum TBA was 129 µmol/L (95% CI: 117.10–140.90) and 163 µmol/L (95% CI: 139.44–186.56) for the success and nonfunctioning groups, respectively (Supporting Information S1: Figure S6, *p* = 0.01). At the 1–4 year interval post‐HPE, the pooled serum TBA was 92.47 µmol/L (95% CI: 75.85–109.095) and 81.75 µmol/L (95% CI: 71.02–92.48) for the success and nonfunctioning groups, respectively (Supporting Information S1: Figure S6, *p* = 0.29). For the >5 year interval post‐HPE, the pooled serum TBA was 27.55 µmol/L (95% CI: 20.35–34.75) and 69 µmol/L (95% CI: 56.26–81.74) for the success and nonfunctioning groups, respectively (Supporting Information S1: Figure S6, *p* < 0.001).

### 3.7 MMP7

The study by Wu et al. in our analysis was the only one that reported the impact of elevated MMP‐7 in BA patients post‐HPE, indicating that patients with elevated MMP‐7 > 10.3 ng/mL (1–4 years after HPE) had a significantly increased risk of non‐function with an HR of 4.29 (95% CI: 1.12–16.52).^39^

### 3.8 Other novel serum markers

A recent review highlighted several novel serum markers for prognostication of patients with BA.^44^ Pro‐inflammatory cytokines are consistently dysregulated: higher IL‐8, IL‐18, and IL‐34 correlate with jaundice, fibrosis, and hepatic dysfunction, whereas higher preoperative IL‐12p40 predicts 3‐month jaundice‐free survival (PPV 81%, NPV 83%) and ~4‐fold better 3‐year native‐liver survival. Extracellular matrix and chaperone markers also carry prognostic information: elevated cartilage oligomeric matrix protein (COMP) and reduced clusterin (CLU) identifies higher‐risk patients after HPE (AUC 0.85; sensitivity 81.5%; specificity 73.5%). Bile‐acid–regulated fibroblast growth factor 19 (FGF‐19) relates to HPE outcomes; levels <109 pg/mL predict reduced native‐liver survival (HR 4.31; 95% CI: 1.90–9.74) and correlate with primary bile acid levels. Finally, the glyco‐marker Mac‐2 binding protein glycan isomer (M2BPGi) reflects fibrosis severity, identifying ≥F3 fibrosis and cirrhosis with high accuracy (cutoff 1.84 COI; AUC 0.93; sensitivity 91%; specificity 96%).^44^

### 3.9 Quality assessment

Quality assessment of studies is provided in Supporting Information S1: Table S3. Most studies had appropriate cohort selection, including representativeness of the inpatient cohort. The most common limitation observed was retrospective cohort study design (all 30 studies), leading to temporal ambiguity between exposure and outcome, reliance on existing records with potential misclassification and missing data, limited ability to track changes in exposure over time, and incomplete or inconsistently reported loss to follow‐up. Although outcomes were clearly defined and valid in most studies, ascertainment methods for those outcomes varied. Finally, several studies used limited single‐center retrospective datasets, which have inherent limitations, including missing or incomplete data on diagnosis of BA thus increasing risk of ascertainment bias.

## 4 DISCUSSION

Our study's literature search identified 30 inclusion studies from an initial pool of 4507 to evaluate serum markers in BA patient outcomes post‐HPE. Our findings revealed a significant distinction in serum markers between successful and non‐functioning HPE outcomes in BA. Serum TB levels were elevated at various postoperative intervals, ALT and GGT were predictive of HPE outcome, and the identification of the novel role of MMP‐7 and TBA levels as prognostic indicators. This study underscores the critical timing of HPE indicating HPE at younger age is associated with long‐term success.

The successful HPE group had an average time to HPE of 63.3 ± 0.18. Several studies reported an earlier meantime of around 45 days, especially among the patients with successful HPE, however these studies had wider confidence intervals and smaller sample sizes.^29^, ^39^, ^45^ The meta‐analysis by Yang et al. showed that BA patients with NLS who underwent HPE at 60 days of age or younger had significantly improved outcomes compared to patients who underwent HPE aged 61–90 days (OR: 1.41, 95% CI: 1.18–1.68).^46^ Our results add to the growing body of evidence showing that HPE success is more likely when performed earlier.

TB levels at the various defined time points after HPE exhibited distinct cutoff values associated with success. TB < 2 mg/dL at 3 months has been widely used as a negative prognostic indicator for BA in pediatric hepatology.^47^ Our results support this data and further add additional time‐points for assessing TB among BA patients. At the 1–3 month time interval, post‐HPE success was associated with a TB of around 2.4 mg/dL. Beyond the 1–3 month postoperative period, means for TB diverged at 4–12 months, but effect sizes (HR/OR ≈ 1.0) indicate that a 12‐month TB < 1 mg/dL is exploratory, not a validated prognostic cutoff. Prospective evaluation within longitudinal risk models is warranted to further explore this 1‐year cut‐off for TB.^47^, ^48^ Based on this finding, longitudinal monitoring with TB measurement at 3 months as well as 12 months may be useful in predicting NLS post‐HPE.

GGT was significantly lower among successful post‐HPE patients at most time intervals. Koga et al. demonstrated significantly lower GGT levels at the 1–3 months in patients with NLS when compared to transplanted patients.^49^ Our study confirmed this finding, while also showing that patients with HPE nonfunctioning had significantly greater GGT levels at 1–3 months (>600 IU/L) and 4–12 months (>450 IU/L). Others have also shown that GGT levels >550 IU/L at 5 months post‐HPE (HR: 1.74, 95% CI: 1.40–2.87) were associated with worse prognosis even in the absence of jaundice.^24^ Our findings support the use of GGT with gradually decreasing cutoffs at different time points as possible prognostic indicators.

Our study highlighted significantly lower ALT levels, of approximately 100 IU/L, within the 1–3 month period post successful HPE, suggesting its possible use as a prognostic marker. Overall, there is a noticeable lack of reporting of post‐HPE ALT levels. This may be due to elevations associated with other complications such as cholangitis and sepsis. However, given the narrow range of ALT as a successful prognostic marker, its use and reporting should be further studied.

At more than 5 years post‐HPE, TBA was shown to be a useful prognostic indicator for success. The main limitation of defining successful post‐HPE cutoff values for TBA lies with the limited number of studies available. Despite the paucity of data, TBA has shown promise as a useful biomarker for assessing the success of HPE even among patients with normalized bilirubin.^8^ TBA can remain elevated in patients with normalized TB, indicating ongoing bile acid congestion. Even after successful HPE, complete improvement in bile acid excretion and metabolism may not be achieved.^50^ Elevated TBA in successful HPE patients with normalized TB suggests that TBA could be a more sensitive prognostic indicator than TB.^50^

MMP‐7, another novel marker in BA, is a matrix metalloproteinase that increases in BA patients with progressive hepatic fibrosis.^51^ As of now, MMP‐7 shows promise as a prognostic indicator at the 1–4 year time point.^39^ More data are required to delineate its use.

This study offers a long‐term protocol with a clear timeline and universal use in assessing BA patients post‐HPE. While the use of TB < 2 mg/dL at 3 months for prognosis has been previously established in the literature, our study identified new values and cut‐offs that could be incorporated into a protocol to guide pediatric hepatologists post‐HPE. This is especially useful in the setting of BA patients with successful HPE that still end up requiring a transplant later in life (Supporting Information S1: Figure S7). Analyzing the magnitude of change in selected serum values from the time of HPE can offer a clearer understanding of both cutoff values and the required changes to predict the success of HPE. This insight can facilitate the establishment of follow‐up protocols with tailored cutoffs for BA patients post‐HPE.

One of the important factors to consider is the strength of variable associations with NLS to help delineate a cost‐effective and practical care process model for post‐HPE surveillance monitoring. There were some studies which explored two or more of the variables enabling some inferences from these data. Venkat et al showed that among TB, GGT, and ALT; TB had the highest significant HR of 2.64 (*p* < 0.001) in predicting the probability of sentinel events among BA patients compared to GGT and ALT, respectively.^36^

Our study highlights several limitations in BA research. First, the absence of unified national datasets underscores the need for multicenter studies. Second, the lack of randomized control trials and small sample sizes in retrospective studies. Third, inconsistent definitions of post‐HPE success, such as jaundice‐free survival or transplantation, call for standardized outcome measures. Fourth, the scarcity of data on novel prognostic indicators like MMP‐7 and TBA necessitates further studies with long‐term follow‐up. Fifth, BA management, specifically HPE with LT as needed, has not changed much for decades and remains the standard of care; thus, older cohorts were included, and the results largely reflect longstanding practice. Sixth, the absence of phenotypic categorization hinders identifying prognostic markers for different BA subtypes. Lastly, standardized protocols for labs and imaging are essential to ensure consistent data collection. This comprehensive identification of gaps lays a structured roadmap for future research directions in the field of BA (Table 2).

**Table 2:** Overview of gaps in the literature with proposed futured directions.

Subject/topic	Gaps in the literature	Steps forward
Data sources	No unified national datasets	Funding multicenter studies to build national registries for BA patients
	No proper characterization of post‐HPE success	Refinement of success post‐HPE whether it should be defined as jaundice free survival, cirrhosis, transplant, or death
Study design	Paucity of data on specific serum values such as MMP‐7 and TBA as prognostic indicators for NLS	Novel values may show promise in assessing prognosis of BA and thus new studies require follow‐up of BA patients with these values being assessed
	Paucity of longitudinal data for BA patients post‐HPE	Requirement of standardized follow‐up post‐HPE (especially when successful) to allow for monitoring the point at which failure may occur
	Paucity of data assessing required change (delta) in serum values post‐HPE	With the establishment of longitudinal follow‐up, the assessment of degree of specific serum values at different time points will be possible. Creating possible new metrics for prognostic indications
Characterizing patient population	Defining the patient population by clearly having standardized labs and imaging	Creating standardized follow‐up time points for post‐HPE Standardizing which serum labs are requested at different stand points Making sure all values collected are following uniform SI units and calculations
	Lack of clear phenotypic categorization in BA studies, which impedes the identification of specific prognostic markers for different BA subtypes or phenotypes	Future research should focus on accurately classifying BA patients by phenotype and investigating whether these subgroups exhibit distinct outcomes, thus enabling the development of tailored treatment approaches and improving prognostic accuracy

Abbreviations: BA, biliary atresia; HPE, hepatoportoenterostomy; MMP‐7, matrix metalloproteinase 7; NLS, native liver survival; TBA, total bile acids.

## 5 CONCLUSION

In conclusion, our study suggests the feasibility of applying serum markers at different time frames post‐HPE as prognostic tools for assessing success. Tracking both absolute cutoffs and significant fluctuations in these values over time will enhance the precision in predicting HPE outcomes. This study serves as a guidepost for clinicians to monitor these specific serum values at predetermined intervals to detect patients at risk of non‐function post‐HPE allowing for timely and appropriate interventions.

## CONFLICT OF INTEREST STATEMENT

The authors declare no conflicts of interest.

## Supporting information


**Supplemental Figure 1:** Pooled hazards ratio (HR) for the association of increased total bilirubin (TB) (mg/dL) post‐HPE on NLS among BA patients * TB: Total Bilirubin, HPE: Hepatoportoenterostomy, NLS: Native Liver survival, BA: Biliary Atresia. **Supplemental Figure 2:** ALT (IU/L) serum values of the studies included stratified by success and non‐function post‐HPE among BA patients *ALT: Alanine Transaminase, HPE: Hepatoportoenterostomy, BA: Biliary Atresia. **Supplemental Figure 3:** Pooled odds ratio (OR) and hazards ratio (HR) for the association of increased ALT (mg/dL) post‐HPE on NLS among BA patients *ALT: Alanine Transaminase, HPE: Hepatoportoenterostomy, NLS: Native Liver survival, BA: Biliary Atresia. **Supplemental Figure 4:** GGT (IU/L) serum values of the studies included stratified by success and non‐function post‐HPE among BA patients *GGT: Gamma‐glutamyl transferase, HPE: Hepatoportoenterostomy, BA: Biliary Atresia. **Supplemental Figure 5:** Pooled odds ratio (OR) and hazards ratio (HR) for the association of increased GGT (TB) (mg/dL) post‐HPE on NLS among BA patients *GGT: Gamma‐glutamyl transferase, HPE: Hepatoportoenterostomy, NLS: Native Liver survival, BA: Biliary Atresia. **Supplemental Figure 6:** TBA (µmol/L) serum values of the studies included stratified by success and non‐function post‐HPE among BA patients *TBA: Total bile acids, HPE: Hepatoportoenterostomy, BA: Biliary Atresia. **Supplemental Figure 7:** Proposed timeline for BA follow‐up to assess for prognosis post‐HPE *HPE: Hepatoportoenterostomy, BA: Biliary Atresia. **Supplemental Table 1:** Studies characterizing serum values as prognostic indicators in BA patients with NLS.*BA: Biliary Atresia, NLS: Native Liver Survival. **Supplemental Table 2:** Pooled OR and HR for the impact of increased serum values on NLS of BA patients included in the meta‐analysis. *OR: Odds Ratio, HR: Hazards Ratio, NLS: Native Liver Survival, HPE: Hepatoportoenterostomy, BA: Biliary Atresia, ALT: Alanine Transaminase, Alk Phos: Alkaline Phosphatase, GGT: Gamma‐glutamyl Transferase, n/a: not available. **Supplemental Table 3:** Quality assessment of studies included in the meta‐analysis. *NA: Not Available, Overall score: Total score out of 14 for each paper using the NIH Quality Assessment Tool for Observational Cohort and Cross‐sectional Studies.

Supplemental_Methods.
