# Supplementary material for: Serum predictors of native liver survival post‐Kasai: Systematic review and meta‐analysis
Source: J Pediatr Gastroenterol Nutr. 2026 Jan 29;82(4):939–48. doi: 10.1002/jpn3.70355 (PMC13050805; doi:10.1002/jpn3.70355)
Supplement: Supplementary file 1 — Supplemental Figure 1: Pooled hazards ratio (HR) for the association of increased total bilirubin (TB) (mg/dL) post‐HPE on NLS among BA patients * TB: Total Bilirubin, HPE: Hepatoportoenterostomy, NLS: Native Liver survival, BA: Biliary Atresia. Supplemental Figure 2: ALT (IU/L) serum values of the studies included stratified by success and non‐function post‐HPE among BA patients *ALT: Alanine Transaminase, HPE: Hepatoportoenterostomy, BA: Biliary Atresia. Supplemental Figure 3: Pooled odds ratio (OR) and hazards ratio (HR) for the association of increased ALT (mg/dL) post‐HPE on NLS among BA patients *ALT: Alanine Transaminase, HPE: Hepatoportoenterostomy, NLS: Native Liver survival, BA: Biliary Atresia. Supplemental Figure 4: GGT (IU/L) serum values of the studies included stratified by success and non‐function post‐HPE among BA patients *GGT: Gamma‐glutamyl transferase, HPE: Hepatoportoenterostomy, BA: Biliary Atresia. Supplemental Figure 5: Pooled odds ratio (OR) and hazards ratio (HR) for the association of increased GGT (TB) (mg/dL) post‐HPE on NLS among BA patients *GGT: Gamma‐glutamyl transferase, HPE: Hepatoportoenterostomy, NLS: Native Liver survival, BA: Biliary Atresia. Supplemental Figure 6: TBA (µmol/L) serum values of the studies included stratified by success and non‐function post‐HPE among BA patients *TBA: Total bile acids, HPE: Hepatoportoenterostomy, BA: Biliary Atresia. Supplemental Figure 7: Proposed timeline for BA follow‐up to assess for prognosis post‐HPE *HPE: Hepatoportoenterostomy, BA: Biliary Atresia. Supplemental Table 1: Studies characterizing serum values as prognostic indicators in BA patients with NLS.*BA: Biliary Atresia, NLS: Native Liver Survival. Supplemental Table 2: Pooled OR and HR for the impact of increased serum values on NLS of BA patients included in the meta‐analysis. *OR: Odds Ratio, HR: Hazards Ratio, NLS: Native Liver Survival, HPE: Hepatoportoenterostomy, BA: Biliary Atresia, ALT: Alanine Transaminase, Alk Phos: Alk [file JPN3-82-939-s001.docx]

**Supplemental Table 1:** Studies characterizing serum values as prognostic indicators in BA patients with NLS

| **Author/Year** | **Study Design** | **Study Database** | **Study Period** | **N (Success (S) & Non-Function (F))** |
| --- | --- | --- | --- | --- |
| **Abdel-Aziz et. al, 2019** | Retrospective cohort | Egypt Menoufia University | N/A | 107 (S: 29 & F: 78) |
| **Abenami et. al, 2023** | Retrospective cohort | Saudi Arabia Multicenter study | 2000 – 2018 | 134 |
| **Capparelli et. al, 2021** | Retrospective cohort | J.P. Garrahan Hospital | January 2008 – May 2018 | 58 (S: 35 & F: 23) |
| **Caruso et. al, 2021** | Retrospective cohort | University of Naples Pediatric Unit | January 2012 – December 2019 | 24 (S: 12 & F: 12) |
| **Cetlik et. al, 2021** | Retrospective cohort | Ege University Faculty of Medicine | January 2005 – December 2018 | 53 (S: 38 & F: 15) |
| **Chung et. al, 2021** | Retrospective cohort | Three centers in Hong Kong | 1980 – 2017 | 231 (S: 153 & F: 70) |
| **Chuslip et. al, 2016** | Retrospective cohort | Thai University Hospitals | 2000 – 2014 | 126 (S: 68 & F: 58) |
| **Ferreira et. al, 2019** | Retrospective cohort | Hospital das Clínicas-Minas Gerais Federal University | 1979 – 2008 | 117 (S: 39 & F: 78) |
| **Ge et. al, 2020** | Retrospective cohort | Tianjin First Central Hospital | July 2003 – July 2018 | 200 (S: 121 & F: 79) |
| **Goda et. al, 2013** | Retrospective cohort | Osaka Japan Medical Center | 1990 – 2010 | 54 (S: 24 & F: 30) |
| **Grieve et. al, 2013** | Retrospective cohort | Kings College Hospital | January 1999 – December 2010 | 260 |
| **Huang et. al, 2020** | Retrospective cohort | National Taiwan University Hospital | August 2000 – April 2019 | 90 (S: 51 & F: 39) |
| **Hukkinen et al** | Retrospective cohort | Helsinki University Children’s Hospital | 1990 – 2016 | 41 (S: 19 & F: 22) |
| **Hwang et al** | Retrospective cohort | Asan Medical Center | August 2014 – March 2020 | 63 (S: 15 & F: 48) |
| **Ihn et al** | Retrospective cohort | Severance Children's Hospital | January 2004 – July 2015 | 169 (S: 125 & 44) |
| **Kim et al** | Retrospective cohort | Sungkyunkwan University School | January 2000 – August 2020 | 90 (S: 43 & F: 47) |
| **Kong et al** | Retrospective cohort | Sichaun University | January 2015 – December 2020 | 205 (S: 104 & F: 101) |
| **Kumar et al** | Retrospective cohort | New Dehli Tertiary Care Liver Institute | January 2010 – January 2018 | 107 (S: 29 & F: 50) |
| **Liu et al** | Retrospective cohort | Beijing Friendship Hospital | January 2017 – December 2019 | 197 (S: 24 & F: 130) |
| **Matcovici et al** | Retrospective cohort | Kings College Hospital | January 2012 – December 2018 | 90 (S: 70 & F: 20) |
| **Nightingale et al** | Retrospective cohort | Royal Alexandra Hospital, Sydney Children’s Hospital, The Hospital for Sick Children | January 1986 – June 2009 | 217 (S: 119 & F: 98) |
| **Qi et al** | Retrospective cohort | Tertiary Pediatric center in Northeast China | January 2011 – June 2021 | 151 (At 2 years: S: 113 & F: 38) (At 5 years S: 22 & F: 63) |
| **Qiao et al** | Retrospective cohort | China United Family Hospital | May 2005 – January 2012 | 244 (S at 6 month 211, 1 year 148, 2 year 100, 3 years 40, 4 years 15) |
| **Qishti et al** | Retrospective cohort | Dr. Sardjito Hospital | June 2012 – April 2018 | 29 (S: 15 & F: 14) |
| **Rodeck et al** | Retrospective cohort | Marienhospital Osnabruck | July 1978 – December 1998 | 24 |
| **Subramaniam et al** | Retrospective cohort | Booth Hall Childrens Hospital | 1976 – 1996 | 49 (S: 27 & F: 22) |
| **Sun et al** | Retrospective cohort | Childrens Hospital Fudan University | January 2012 – December 2017 | 1259 (S: 428 & F: 665) |
| **Venkat et al** | Retrospective cohort | ChiLDReN database | June 2004 – August 2017 | 240 (S: 202 & F: 38) |
| **Wang et al** | Retrospective cohort | Sun Yat-sen University | February 2016 – October 2020 | 102 |
| **Witt et al** | Retrospective cohort | University Medical Center Groningen | January 1987 – June 2015 | 100 (S: 57 & F: 43) |
| **Wu et al** | Retrospective cohort | Taiwan University Hospital | January 2008 – April 2018 | 36 |
| **Yanchar et al** | Retrospective cohort | 2 pediatric surgical centers in Alberta | September 1980 – September 1994 | 23 (S: 7 & F: 16) |

^*^ BA: Biliary Atresia, NLS: Native Liver Survival

**Supplemental Table 2:** Pooled OR and HR for the impact of increased serum values on NLS of BA patients included in the meta-analysis.

| **Serum Values** | **Serum Values** | **1-3 months** | **4-12 months** | **1-4 years** | **>5 years** |
| --- | --- | --- | --- | --- | --- |
| **Total Bilirubin**  **(95%CI) mg/dL** | **HR** | n/a | 0.94 (0.56-1.57) | 1.00 (0.99-1.01) | 0.99 (0.99-1.01) |
|  | **OR** | 1.19 (1.09-1.29) | 3.47 (2.15-5.60) | 1.02 (1.01-1.03) | n/a |
| **Direct Bilirubin**  **(95%CI) mg/dL** | **HR** | n/a | n/a | 1.00 (0.99-1.01) | 0.99 (0.99-1.00) |
|  | **OR** | 1.13 (1.03-1.25) | n/a | n/a | n/a |
| **Alanine Transaminase**  **(95%CI)** | **HR** | n/a | n/a | 0.99 (0.99-1.01) | n/a |
|  | **OR** | n/a | n/a | 0.99 (0.98-0.99) | n/a |
| **Gamma-glutamyl Transferase**  **(95%CI) IU/L** | **HR** | n/a | n/a | 1.56 (1.31-1.86) | n/a |
|  | **OR** | n/a | 1.74 (1.22-2.48) | n/a | n/a |

^*^ OR: Odds Ratio, HR: Hazards Ratio, NLS: Native Liver Survival, HPE: Hepatoportoenterostomy, BA: Biliary Atresia, ALT: Alanine Transaminase, Alk Phos: Alkaline Phosphatase, GGT: Gamma-glutamyl Transferase, n/a: not available

**Supplemental Table 3:** Quality assessment of studies included in the meta-analysis.

| **Author/Year** | **1** | **2** | **3** | **4** | **5** | **6** | **7** | **8** | **9** | **10** | **11** | **12** | **13** | **14** | **Overall Score** |
| --- | --- | --- | --- | --- | --- | --- | --- | --- | --- | --- | --- | --- | --- | --- | --- |
| **Abdel-Aziz et. al, 2019** | Yes | Yes | NA | Yes | No | Yes | Yes | No | Yes | No | Yes | NA | NA | No | 7 |
| **Abenami et. al, 2023** | Yes | Yes | NA | Yes | No | Yes | Yes | No | No | No | Yes | NA | NA | Yes | 7 |
| **Capparelli et. al, 2021** | Yes | Yes | NA | Yes | No | Yes | Yes | No | Yes | No | Yes | NA | NA | No | 7 |
| **Caruso et. al, 2021** | Yes | Yes | NA | Yes | No | Yes | Yes | No | No | No | Yes | NA | NA | No | 6 |
| **Cetlik et. al, 2021** | Yes | Yes | NA | Yes | No | Yes | Yes | No | Yes | No | Yes | NA | NA | Yes | 8 |
| **Chung et. al, 2021** | Yes | Yes | NA | Yes | No | Yes | Yes | No | Yes | No | Yes | NA | NA | No | 7 |
| **Chuslip et. al, 2016** | Yes | Yes | NA | Yes | No | Yes | Yes | No | Yes | Yes | Yes | NA | NA | Yes | 9 |
| **Ferreira et. al, 2019** | Yes | Yes | NA | Yes | No | Yes | Yes | No | Yes | No | Yes | NA | NA | Yes | 8 |
| **Ge et. al, 2020** | Yes | Yes | NA | Yes | No | Yes | Yes | Yes | Yes | Yes | Yes | NA | NA | No | 9 |
| **Goda et. al, 2013** | Yes | Yes | NA | Yes | No | Yes | Yes | No | Yes | No | Yes | NA | NA | No | 7 |
| **Huang et. al, 2020** | Yes | Yes | NA | Yes | No | Yes | Yes | No | Yes | No | Yes | NA | NA | Yes | 8 |
| **Hukkinen et al** | Yes | Yes | NA | Yes | No | Yes | Yes | No | Yes | Yes | Yes | NA | NA | Yes | 9 |
| **Hwang et al** | Yes | Yes | NA | Yes | No | Yes | Yes | Yes | Yes | No | Yes | NA | NA | No | 7 |
| **Ihn et al** | Yes | Yes | NA | Yes | No | Yes | Yes | No | No | No | Yes | NA | NA | Yes | 7 |
| **Kim et al** | Yes | Yes | NA | Yes | No | Yes | Yes | No | Yes | No | Yes | NA | NA | No | 7 |
| **Kong et al** | Yes | Yes | NA | Yes | No | Yes | Yes | No | Yes | No | Yes | NA | NA | Yes | 8 |
| **Kumar et al** | Yes | Yes | NA | Yes | No | Yes | Yes | No | Yes | Yes | Yes | NA | NA | No | 7 |
| **Liu et al** | Yes | Yes | NA | Yes | No | Yes | Yes | Yes | Yes | No | Yes | NA | NA | No | 8 |
| **Matcovici et al** | Yes | Yes | NA | Yes | No | Yes | Yes | No | Yes | No | Yes | NA | NA | Yes | 8 |
| **Nightingale et al** | Yes | Yes | NA | Yes | No | Yes | Yes | No | Yes | No | Yes | NA | NA | Yes | 8 |
| **Qi et al** | Yes | Yes | NA | Yes | No | Yes | Yes | Yes | Yes | Yes | Yes | NA | NA | Yes | 10 |
| **Qiao et al** | Yes | Yes | NA | Yes | No | Yes | Yes | No | Yes | Yes | Yes | NA | NA | Yes | 9 |
| **Qishti et al** | Yes | Yes | NA | Yes | No | Yes | Yes | No | Yes | No | Yes | NA | NA | Yes | 8 |
| **Rodeck et al** | Yes | Yes | NA | Yes | No | Yes | Yes | No | No | No | Yes | NA | NA | Yes | 7 |
| **Subramaniam et al** | Yes | Yes | NA | Yes | No | Yes | Yes | Yes | Yes | No | Yes | NA | NA | No | 8 |
| **Sun et al** | Yes | Yes | NA | Yes | No | Yes | Yes | No | Yes | No | Yes | NA | NA | Yes | 8 |
| **Venkat et al** | Yes | Yes | NA | Yes | No | Yes | Yes | Yes | Yes | Yes | Yes | NA | NA | Yes | 10 |
| **Wang et al** | Yes | Yes | NA | Yes | No | Yes | Yes | No | No | No | Yes | NA | NA | Yes | 7 |
| **Witt et al** | Yes | Yes | NA | Yes | No | Yes | Yes | No | Yes | No | Yes | NA | NA | No | 7 |
| **Wu et al** | Yes | Yes | NA | Yes | No | Yes | Yes | No | No | No | Yes | NA | NA | Yes | 7 |
| **Yanchar et al** | Yes | Yes | NA | Yes | No | Yes | Yes | Yes | Yes | No | Yes | NA | NA | No | 8 |

^*^NA: Not Available, Overall score: Total score out of 14 for each paper using the NIH Quality Assessment Tool for Observational Cohort and Cross-sectional Studies

**Supplemental Figures:**


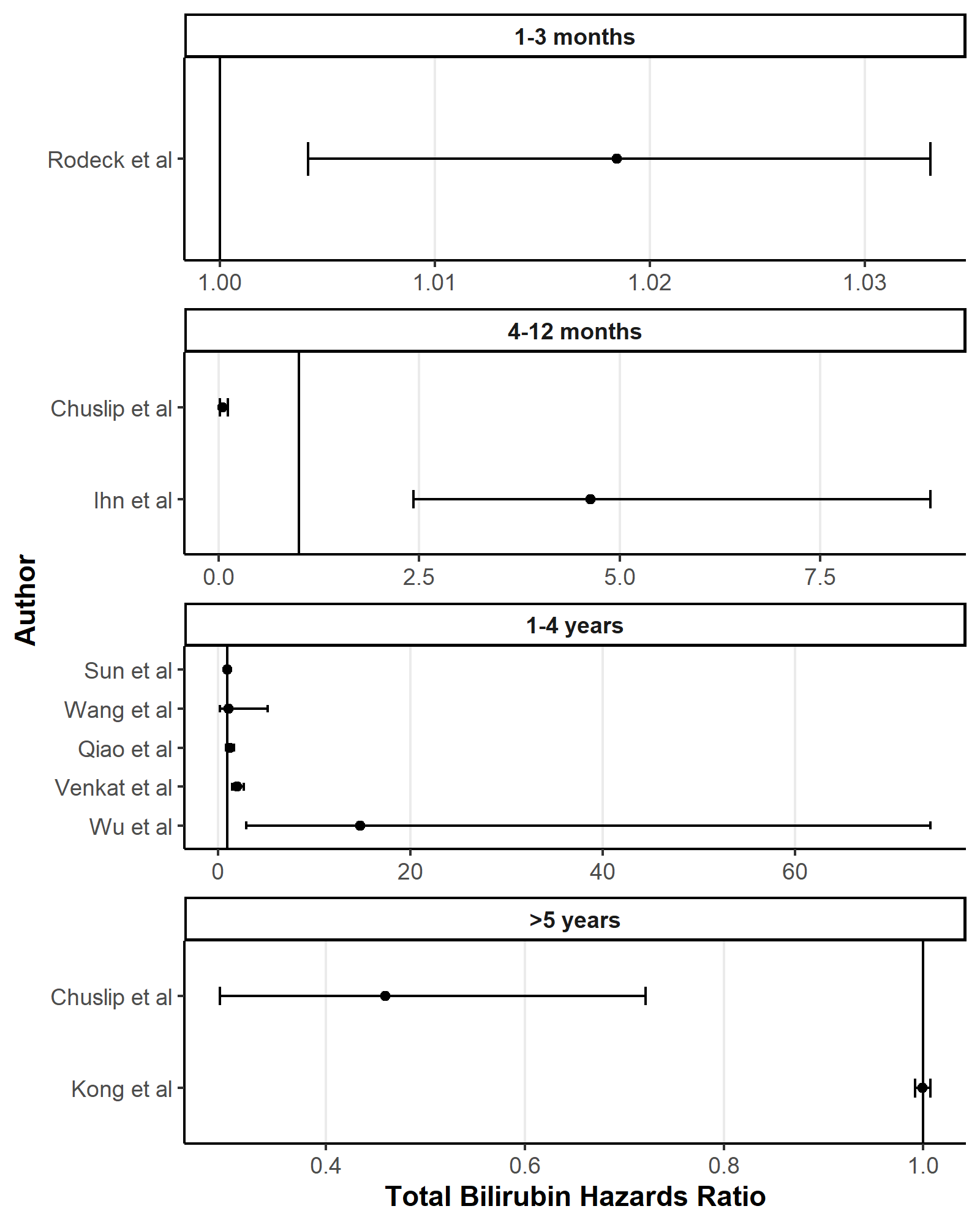


**Supplemental Figure 1:** Pooled hazards ratio (HR) for the association of increased total bilirubin (TB) (mg/dL) post-HPE on NLS among BA patients

^*^ TB: Total Bilirubin, HPE: Hepatoportoenterostomy, NLS: Native Liver survival, BA: Biliary Atresia

**
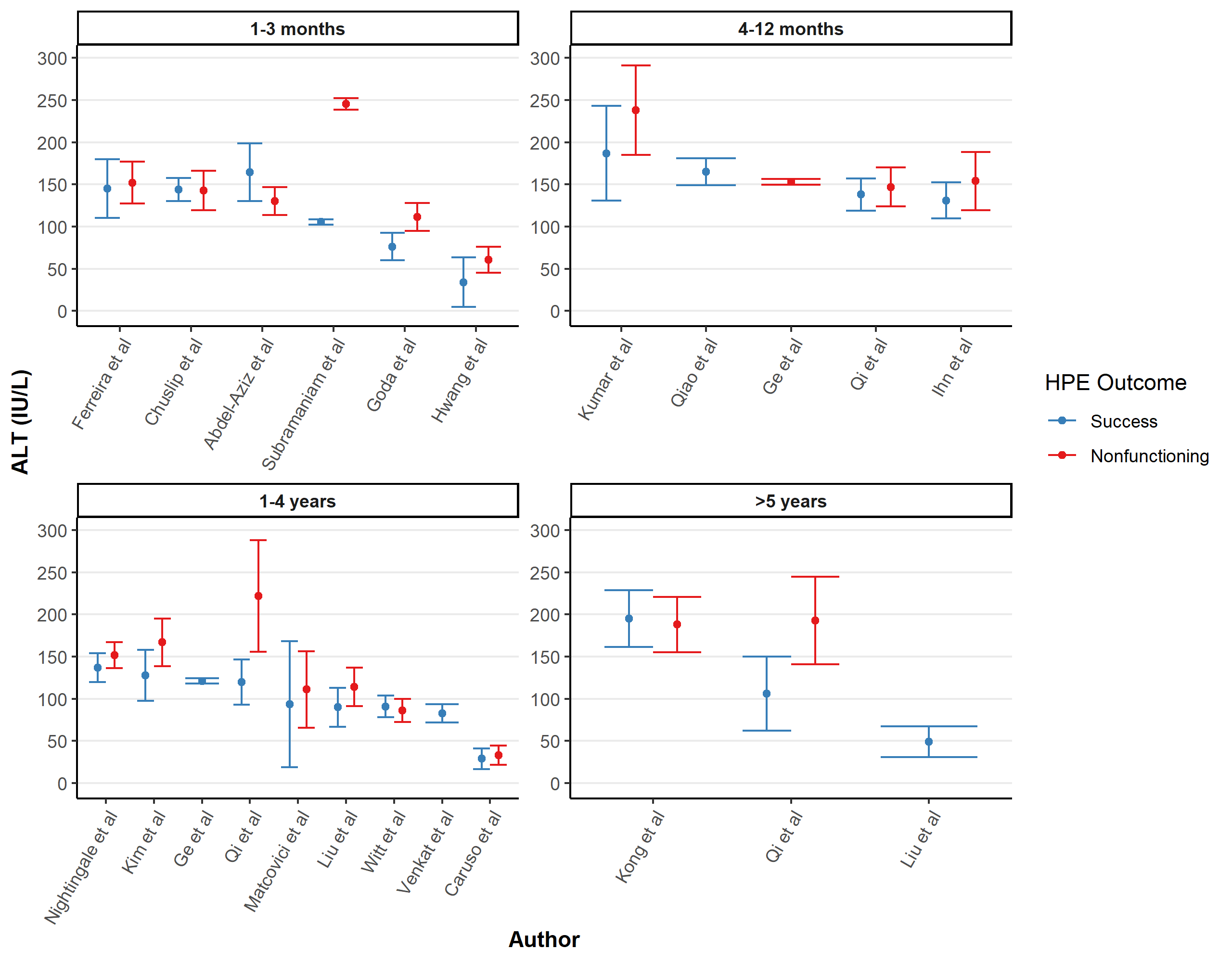
**

**Supplemental Figure 2:** ALT (IU/L) serum values of the studies included stratified by success and non-function post-HPE among BA patients

^*^ ALT: Alanine Transaminase, HPE: Hepatoportoenterostomy, BA: Biliary Atresia

**
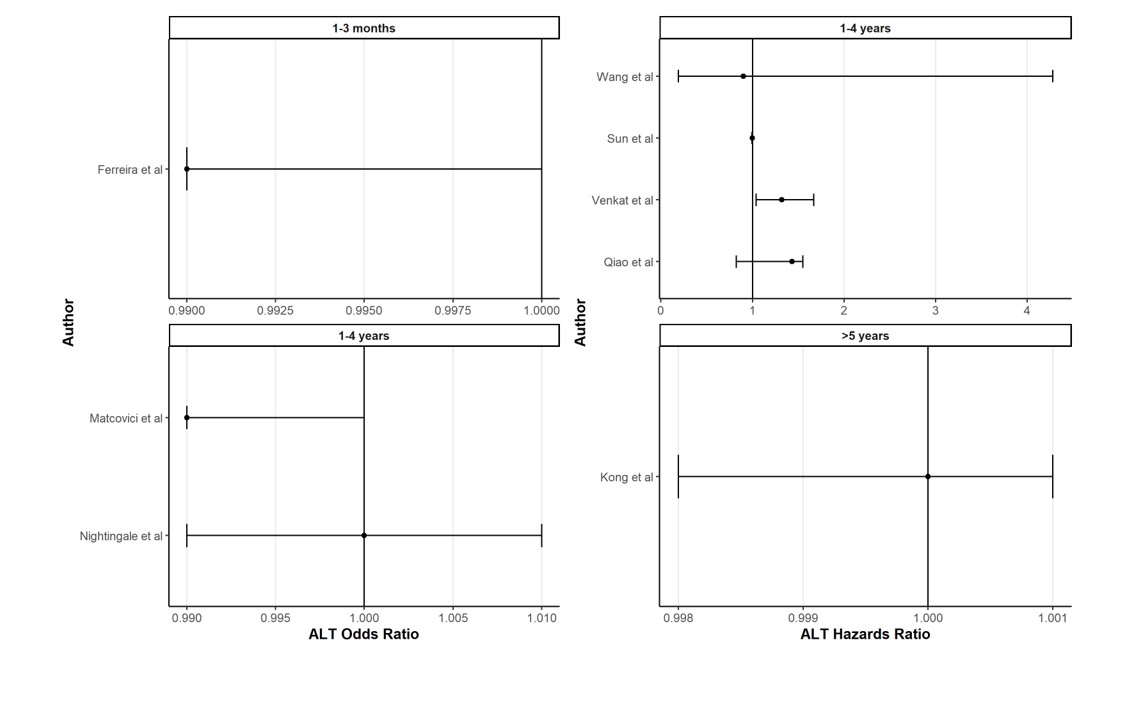
**

**Supplemental Figure 3:** Pooled odds ratio (OR) and hazards ratio (HR) for the association of increased ALT (mg/dL) post-HPE on NLS among BA patients

^*^ ALT: Alanine Transaminase, HPE: Hepatoportoenterostomy, NLS: Native Liver survival, BA: Biliary Atresia

**
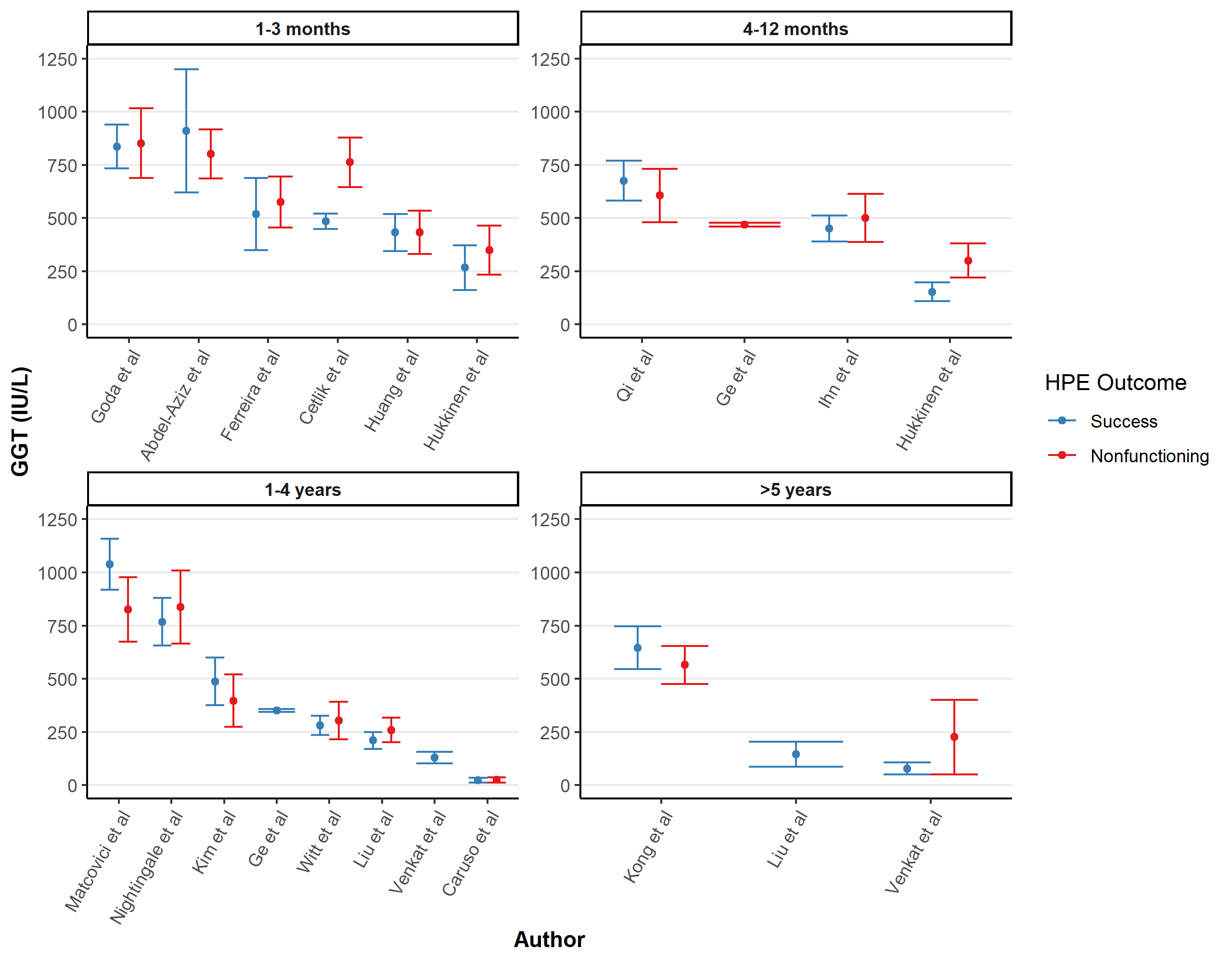
**

**Supplemental Figure 4:** GGT (IU/L) serum values of the studies included stratified by success and non-function post-HPE among BA patients

^*^GGT: Gamma-glutamyl transferase, HPE: Hepatoportoenterostomy, BA: Biliary Atresia

**
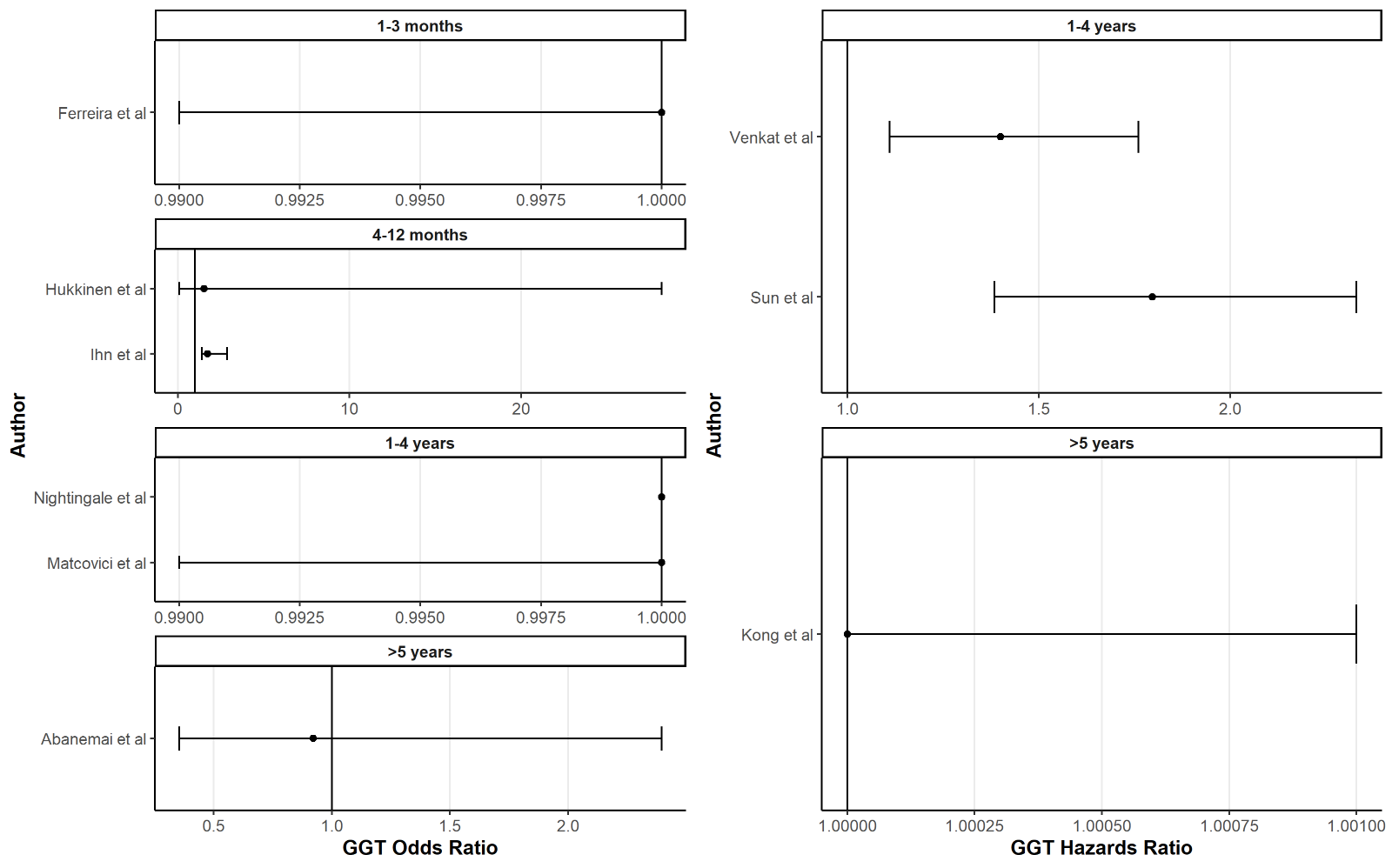
**

**Supplemental Figure 5:** Pooled odds ratio (OR) and hazards ratio (HR) for the association of increased GGT (TB) (mg/dL) post-HPE on NLS among BA patients

^*^GGT: Gamma-glutamyl transferase, HPE: Hepatoportoenterostomy, NLS: Native Liver survival, BA: Biliary Atresia

**
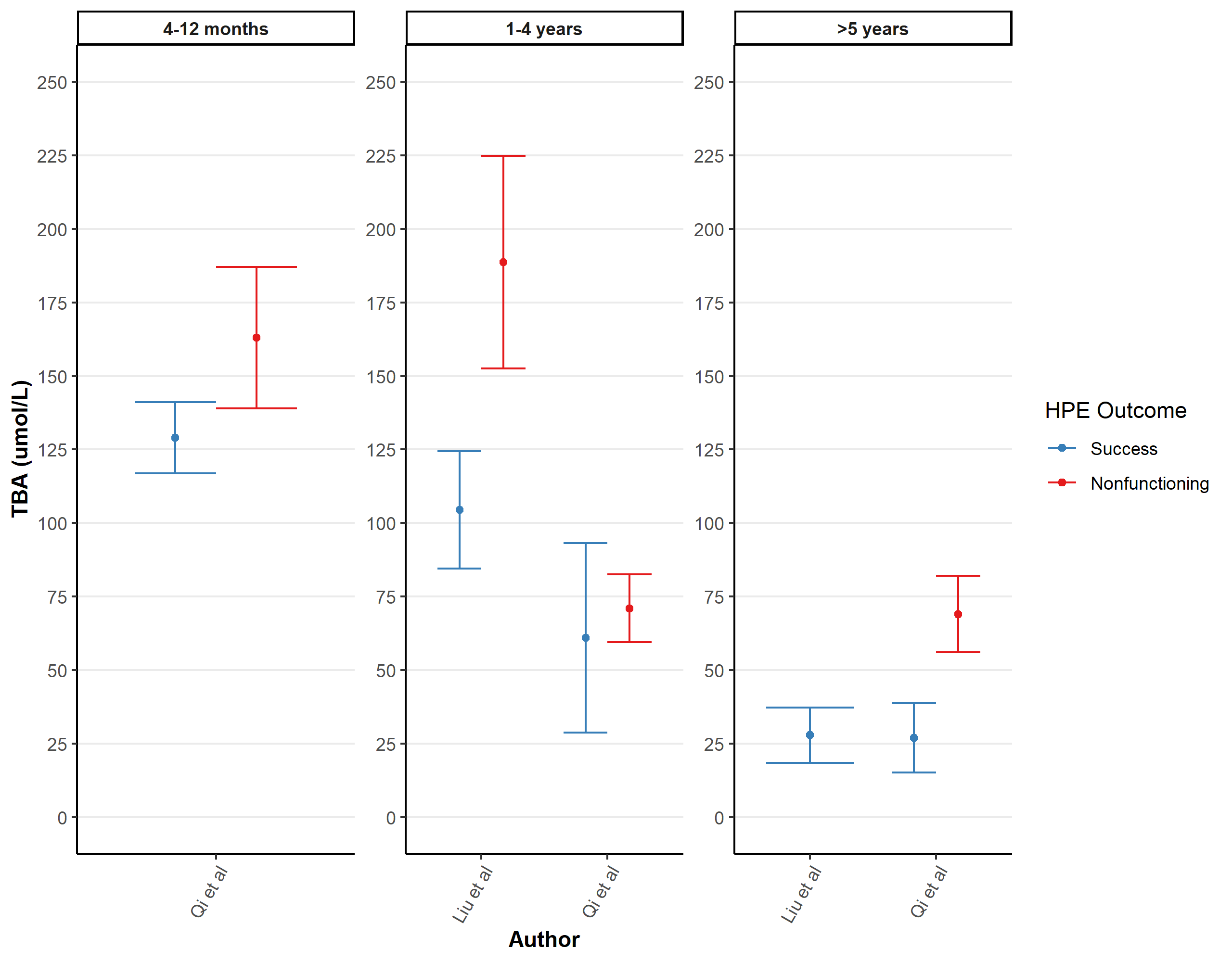
**

**Supplemental Figure 6:** TBA (µmol/L) serum values of the studies included stratified by success and non-function post-HPE among BA patients

^*^TBA: Total bile acids, HPE: Hepatoportoenterostomy, BA: Biliary Atresia

**
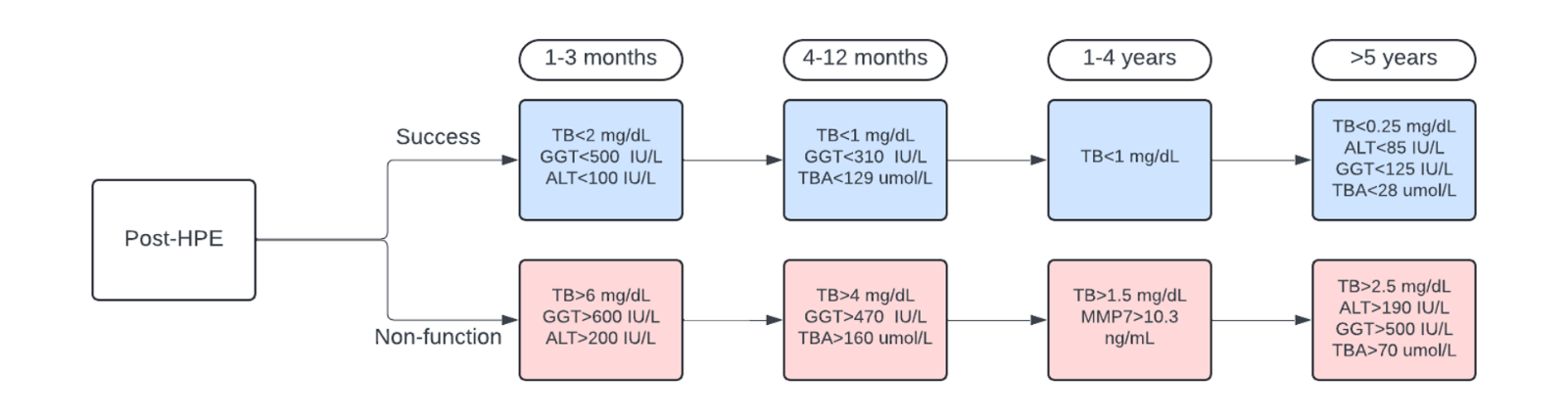
**

**Supplemental Figure 7:** Proposed timeline for BA follow-up to assess for prognosis post-HPE

*HPE: Hepatoportoenterostomy, BA: Biliary Atresia
