# Supplementary material for: Serum predictors of native liver survival post‐Kasai: Systematic review and meta‐analysis
Source: J Pediatr Gastroenterol Nutr. 2026 Jan 29;82(4):939–48. doi: 10.1002/jpn3.70355 (PMC13050805; doi:10.1002/jpn3.70355)
Supplement: Supplementary file 2 — Supplemental_Methods. [file JPN3-82-939-s002.docx]

**Supplemental Methods:**

**Database: OVID Medline ® and Epub Ahead of Print, In-Process, In-Data-Review & Other Non-Indexed Citations and Daily 1946 to December 1, 2023**

1. Exp Biliary Atresia/ 🡪 *3,687*
2. (biliary adj3 atresia).tw. 🡪 *4,757*
3. 1 or 2 🡪 *5,305*
4. (predictors or associations or risk factors).tw. 🡪 *1,084,951*
5. (transplant free survival or long-term survival or survival).tw. 🡪 *1,033,484*
6. Native liver.tw. 🡪 *926*
7. 4 or 5 or 6 🡪 *2,030,869*
8. 3 and 7 🡪 *1,147*

**Embase 1974 to December 1, 2023**

1. ‘biliary atresia’/ 🡪 *6,527*
2. (biliary adj3 atresia).tw. 🡪 *8,036*
3. 1 or 2 🡪 *9,669*
4. (predictors or associations or risk factors).tw. 🡪 *1,795,711*
5. (transplant free survival or long-term survival or survival).tw. 🡪 *1,781,901*
6. Native liver.tw. 🡪 *1,805*
7. 4 or 5 or 6 🡪 *3,407,063*
8. 3 and 7 🡪 *2,380*

**Pubmed Inception to December 1, 2023**

(((“biliary atresia” [MeSH Terms] OR “biliary atresia” [Tital/Abstract])) AND (Predictors OR Risk Factors OR Associations)) AND (transplant free survival or long-term survival OR survival OR native liver) 🡪 *618*

**Scopus Inception to December 1, 2023**

Abstract title, Abstract, Keywords (“biliary atresia”) AND Abstract title, Abstract, Keywords (“predictors” OR “associations” OR “risk factors”) AND Abstract title, Abstract, Keywords (“transplant free survival” OR “long-term survival” OR “survival”) AND Abstract title, Abstract, Keywords (“native liver”) 🡪 *362*
